# Physical and Physiological Characterization of Custom-Made Virtual Reality Exergames: A Pilot Study

**DOI:** 10.3390/sports13110380

**Published:** 2025-11-03

**Authors:** Cíntia França, Hildegardo Noronha, Eva Freitas, Pedro Campos, Rui T. Ornelas, Élvio R. Gouveia

**Affiliations:** 1 Department of Physical Education and Sport, University of Madeira, 9020-105 Funchal, Portugal; rui.ornelas@staff.uma.pt (R.T.O.); erubiog@staff.uma.pt (É.R.G.); 2 LARSyS, Interactive Technologies Institute, 9020-105 Funchal, Portugal; hnoronha@staff.uma.pt (H.N.); evaazevedofreitas@gmail.com (E.F.); 3 WoWSystems Informática Lda, 9050-446 Funchal, Portugal; pedro.campos.pt@gmail.com; 4 CIPER, Faculty of Human Kinetics, University of Lisbon, 1649-004 Lisbon, Portugal; 5 Center for the Interdisciplinary Study of Gerontology and Vulnerability, University of Geneva, 1227 Carouge, Switzerland

**Keywords:** older adults, ageing, heart rate, physical activity, user tests

## Abstract

The continuous growth of the older adult population demands an urgent need to provide innovative ways to stimulate physical activity and promote functional health. This study presents FitFest, a custom-made virtual reality (VR) designed to deliver a complete physical activity (PA) session for older adults. A pilot study involving seven older adults (67.0 ± 3.8 years) was conducted, comprising 18 user testing sessions of two VR exergames: Wine Fest and Flower Fest. PA intensity and heart rate (HR) were measured. The rate of perceived exertion (RPE) and the participants’ rating of the system’s usability were also registered. Overall, sessions involved mostly sedentary behavior (56.5 ± 20.4%) and light PA (42.1 ± 19.3%), with an average of 436.7 steps and 92.1 bpm. Wine Fest elicited less sedentary behavior (53.6 ± 22.1% vs. 62.8 ± 16.2%), higher light PA intensity (44.7 ± 20.4% vs. 36.4 ± 17.0%), and a higher step count (503.0 ± 345.4 vs. 291.0 ± 143.1) than the Flower Fest, although not significantly. Tasks requiring cardiovascular effort and strength were rated as more physically demanding. Most participants found the system user-friendly and showed interest in continued use, though technical support was essential due to limited familiarity with VR. The findings suggest FitFest has potential to promote light PA in older adults, highlighting the importance of usability and support in tech-based interventions.

## 1. Introduction

Ageing is related to declines in physical fitness components, such as strength, power, cardiorespiratory fitness, and balance, leading to physical limitations in daily functional activities [1,2]. Evidence shows that exercise interventions in older adults can improve functional capacity, contributing to better physical health and quality of life [3,4].

According to the World Health Organization’s physical activity (PA) guidelines, older adults should engage in at least 150 min of moderate-intensity or 75 min of vigorous activity per week, emphasizing aerobic capacity [5]. In addition, incorporating muscle-strengthening and balance-enhancing exercises is considered essential for fall prevention, which is a significant and common concern in this population [6,7]. It is estimated that approximately one-third of adults aged 65 years and over living in community settings experience at least one fall annually [8], with nearly half of these individuals falling more than once [7]. Although falls are typically multifactorial, previous research has linked them to impairments in balance, muscle strength, and gait [9,10]. For instance, in a cohort of 619 older adults aged 69.5 years, gait speed and postural balance were inversely associated with fall incidence. Moreover, experiencing falls negatively affected health-related quality of life [6].

There is substantial scientific literature that supports the benefits of PA in reducing the risk of several age-related morbidities and all-cause mortality [1]. Participation in PA programs has been related to a 32% to 40% reduction in the risk of fall-related injuries, primarily through the preservation of physical function and mobility [11]. Furthermore, a systematic review and meta-analysis of longitudinal cohort studies found that higher levels of PA were linked to a 39% increase in the odds of healthy aging [12].

The global growth of the older adult population highlights the urgent need to develop strategies that support maintaining and improving functional health throughout aging. In response to the rapid advancement of technology, exergames (videogames that combine physical exercise with interactive gameplay), have emerged as promising platforms to enhance PA across several populations, including older adults [13,14]. Research using exergames to promote physical fitness components in older populations has reported positive effects on balance [15], strength, and aerobic capacity [16]. Besides physical benefits, exergames have been recognized for their potential to provide mental stimulation, which is crucial to mitigate age-related cognitive declines [17]. A recent study analyzing randomized controlled trials conducted to examine the effects of virtual reality (VR) exergames on cognition and depression in older adults found positive effects for cognitive function and memory [18].

Although a growing body of research has explored the implementation of exergames among older adults, most studies have employed commercial games for Nintendo Wii [15,19] and Xbox Kinect [20,21]. Among the various components of physical fitness targeted in exergaming interventions, balance has received the most attention [10,15,21], likely due to its well-established link to fall prevention. Nonetheless, muscle strength and aerobic capacity are also essential for maintaining functional health and independence in older adults [5], and should not be overlooked.

Overall, commercially available games are not necessarily designed to meet the specific needs of older populations and often fail to deliver structured and progressive gameplay that targets multiple components of physical fitness. On the other hand, these systems often lack precision in motion detection and rely on repetitive movements and static scenarios, which can lead to boredom and affect user motivation over time. Therefore, the current study’s aim is twofold: (1) to present a VR custom-made exergame prototype for older adults (FitFest) designed to deliver a full exercise session tailored for older adults according to PA guidelines for this population; (2) to assess the acute physical and physiological responses elicited during gameplay, and the system’s usability.

## 2. Materials and Methods

### 2.1. Game Development

#### 2.1.1. Game Concept

The FitFest comprises two exergames, composed of five scenarios/mini games each, incorporating sociocultural narratives to create an engaging and enjoyable exercise experience. The prototype was developed by the authors without external contractors, involving a multidisciplinary team of researchers from the fields of human–computer interaction, sports science, and design, and no intellectual property issues need to be acknowledged. Additionally, input from the target population was integrated through qualitative analysis after a focus group data collection to ensure that the prototype aligned with their preferences and needs. The results of qualitative analysis are summarized in a previous study [22]. The custom-made exergames were designed to replicate a complete exercise session, including its typical duration (approximately 45 min) and structure (comprising a warm-up, main phase, and cool-down). Exercise selection was guided by established recommendations for physical fitness training in older adults [23]. Moreover, since aging is commonly associated with a gradual decline in cognitive function [17], the game’s design also integrated cognitive stimuli aimed at enhancing working memory, problem-solving, and constructive thinking skills.

Given the importance placed on social and cultural aspects by the target population [22], each game was designed based on traditional festivities from Madeira Island:

- Wine Fest: It mimics the rich Portuguese tradition of the winemaking process, including the grape picking, sorting, and transport.
- Flower Fest: It replicates a popular celebration in Madeira Island, involving the creation of floral decorations, traditional dance, and participation in a parade.

**Figure 1:**
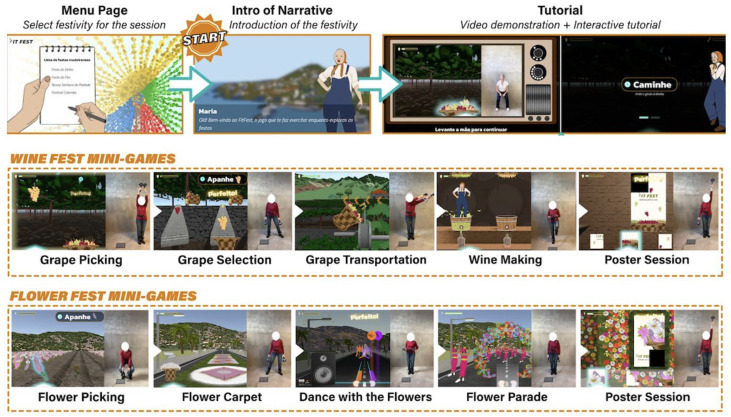
Illustration of the FitFest user interface and scenarios.

A detailed description and illustration of the game scenarios can be found in Table 1 and Table 2, and Figure 1, respectively.

To accommodate the diverse physical and cognitive profiles within the target population, the FitFest was designed with three adjustable difficulty levels: (1) elementary; (2) standard; and (3) advanced. These levels varied in terms of action speed, range of movement, and the complexity of cognitive stimuli, allowing the gameplay to be tailored to individual abilities and needs.

To ensure that FitFest stood out from conventional exergames, a narrative element was integrated to foster a deeper emotional connection between the player and the in-game experience. The storyline follows a 65-year-old Portuguese resident who embarks on a journey to rediscover their youth and escape the monotony of daily routines in new and enriching experiences.

Following the recommendations of previous literature [24], a mandatory tutorial was implemented before each scenario to guarantee a clear understanding of the game mechanics. During the tutorial, players were required to replicate the movements involved in the upcoming activity, ensuring that correct form and posture were understood prior to gameplay. An illustration of the game narrative and tutorials can be seen in Figure 1.

#### 2.1.2. Hardware

**Figure 2:**
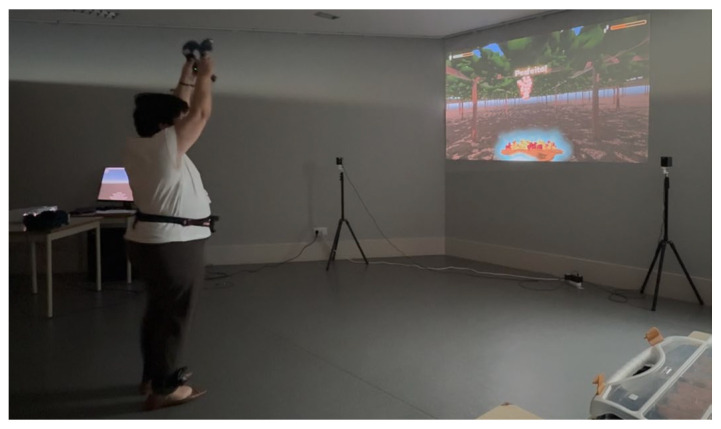
Gameplay illustration.

To accurately track body movements, three HCT Vive trackers were positioned at the participant’s ankles and waist, along with two hand controllers. This setup enables precise tracking of key joint movements in a gaming area approximately 2 m wide. Considering the insights from the previous qualitative analysis regarding technology use and user preferences [22], a wall-projection system (Figure 2) was implemented for the interface. This choice aimed to reduce common concerns associated with headset-based VR systems, such as balance issues, motion sickness, and potential discomfort or fear.

#### 2.1.3. Software

FitFest development was performed using Unity 2022 as the game engine. A 2.5D game approach was adopted, combining 2D objects with 3D scenarios. This design choice enabled efficient development cycles while maintaining visual appeal and incorporating a sense of depth that could be exploited across several scenarios. To design prioritizes intuitiveness and affordance to elicit natural gestures from players. To minimize potential frustration from a population not accustomed to exergames, a short video demonstrates the interaction of gestures with the game, followed by a brief interactive tutorial. When the game starts, the player is familiar with all the required gestures and knows when to use them.

### 2.2. User Tests

A set of user tests was conducted to verify the physical and physiological effects of the FitFest exergames, using PA intensity and heart rate (HR) values. The system’s usability and participants’ rate of perceived exertion were also assessed. The testing sessions took place in a local community center, where participants were recruited and worked with older adults for four weeks. Two members of the research team supervised all testing sessions.

Before the study began, participants’ height was measured using a portable stadiometer (SECA 213, Hamburg, Germany) to the nearest 0.1 cm, and body mass was measured using a portable scale (SECA 760, Hamburg, Germany) to the nearest 0.1 kg. During the testing session, participants were equipped with an accelerometer to examine PA intensity and an HR sensor to monitor heart rate variability. After playing each scenario, participants could rate their perceived exertion directly in the game using a 10-point scale. After the testing session, participants were asked a system usability questionnaire.

#### 2.2.1. Participants

The study included a convenience sample of seven older adults (2 males) aged 67.0 ± 3.8 years. All participants were healthy, actively involved in the local community center activities, and without medical restrictions to exercise engagement. Throughout the study, participants performed a total of 18 user testing sessions, all played at the standard difficulty level. Although the experimental design scheduled one session per week per participant, full compliance was not achieved. Two participants completed all four sessions, two completed three sessions, one completed two sessions, and two attended only one session. All the procedures implemented in this study were approved by the Ethics Committee of the University of Madeira (PARECER No. 111/CEUMA/2024, 25 March). Participation was voluntary, and all the participants had previously signed an informed consent form.

#### 2.2.2. Physical Activity

Physical activity (PA) intensity was assessed using the ActiGraph GT3X+ accelerometer [25]. Participants used the accelerometer during the entire session, which was placed on their right hip. The instrument was initialized with a 30 Hz sampling frequency, and raw data from GT3X+ files were converted to 10s epoch data files before analysis. The time spent in sedentary behavior, light activity, moderate activity, moderate-to-vigorous activity, and vigorous activity was derived using the ActiLife software, version 6 (ActiGraph, Pensacola, FL, USA), using the cutoff points suggested by previous research on older adults [26,27]. The number of steps was also analyzed. The accelerometer was programmed before each session based on the participants’ data, and the data collection started at the beginning of the exergame session.

#### 2.2.3. Heart Rate

During the exergame session, participants were equipped with the Polar H10 sensor to monitor heart rate (HR) responses. The device was positioned in the participants’ chest using the manufacturer’s strap. This positioning ensures optimal contact with the skin and allows the sensor’s electrodes to collect real-time HR data.

#### 2.2.4. Rate of Perceived Exertion

The rate of perceived exertion (RPE) scale [28] was incorporated into the exergame after each scenario conclusion. Using the 10-point scale (0—extremely easy, 10—extremely hard), participants had to move to the right or left side to indicate the number that would better correspond to their effort. After choosing the number that would fit their effort level, participants had to raise one hand above their head to select the number. Since each exergame comprises five scenarios, this task was done five times during the session.

#### 2.2.5. Usability of the System

The system’s usability was examined using the Portuguese version of the System Usability Scale (SUS) [29]. The instrument consisted of 10 items, alternating between positive and negative statements, and was scored on a 5-point Likert scale (1—strongly disagree, 5—strongly agree). It was completed immediately after the exergame session. Its final score ranges from 0 to 100, where higher scores indicate better usability. The full instrument can be consulted in previous literature [29].

### 2.3. Statistical Analysis

Descriptive statistics are presented as mean ± standard deviation for PA and HR variables. A Mann–Whitney U test was conducted to explore differences between the Wine Fest and the Flower Fest regarding PA intensity, HR, and system usability. Since the structure of targeted physical fitness components of both exergames was similar, RPE was assessed globally to understand which scenarios were more demanding. The statistical analysis and illustration procedures were conducted using IBM SPSS Statistics 29.0 (SPSS Inc., Chicago, IL, USA) and GraphPad Prism (version 10, GraphPad Software, San Diego, CA, USA). The significance level was set at 5%.

## 3. Results

Due to an accelerometer malfunction during data collection, two sessions were excluded from the analysis of PA intensity and HR. As a result, 16 sessions (11 for the Wine Fest and 5 for the Flower Fest) remained for analysis.

Table 3 summarizes the descriptive statistics for PA intensity and HR for all users’ testing sessions. Overall, FitFest exergames predominantly involved sedentary behavior (56.5 ± 20.4%), followed by light PA (42.1 ± 19.3%). A mean value of approximately 436.7 steps and 92.1 bpm was recorded throughout the testing session.

**Figure 3:**
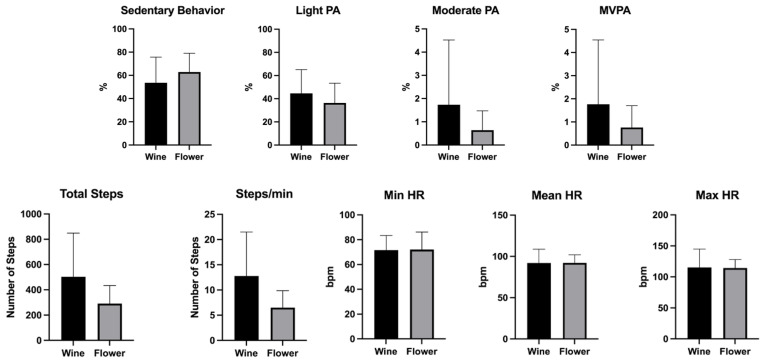
Physical activity intensity, steps, and heart rate analysis of FitFest exergames.

Figure 3 presents physical and physiological responses to the FitFest exergames. The Wine Fest was able to promote less sedentary behavior (53.6 ± 22.1% vs. 62.8 ± 16.2%), higher light PA intensity (44.7 ± 20.4% vs. 36.4 ± 17.0%), and a superior number of total steps (503.0 ± 345.4 vs. 291.0 ± 143.1) than the Flower Fest. However, the differences were not statistically significant. Concerning HR measures, values were similar between both exergames (92.1 bpm).

**Figure 4:**
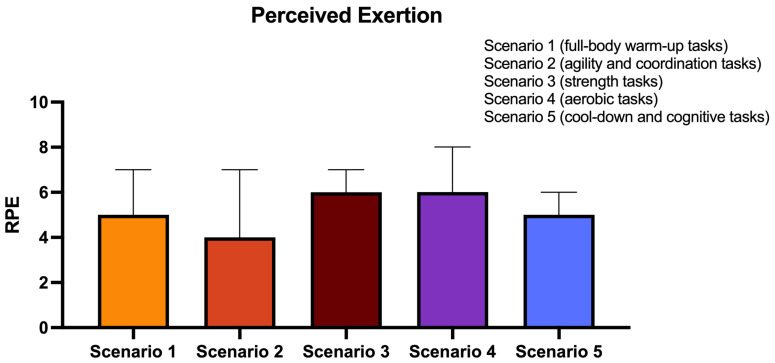
Overall perceived exertion of the FitFest exergames.

The analysis of RPE (Figure 4) showed a higher perceived exertion in scenarios 3 and 4, composed mainly of tasks related to strength and cardiorespiratory fitness. In contrast, scenario 2, which mainly addressed agility and coordination movements, was perceived as less physically demanding.

**Figure 5:**
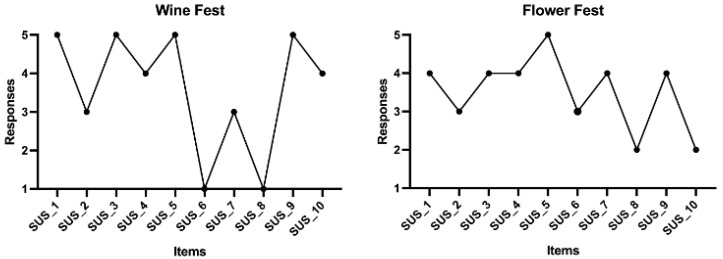
Usability analysis of the Wine Fest and Flower Fest systems.

Regarding the system’s usability (Figure 5), most users indicated that they would like to use the system more frequently and found it easy to use. Users also reported feeling confident while playing and agreed that the system’s functions were well integrated. However, users also agreed that they would need the support of a technician or specialist to use the system effectively. When comparing exergames’ usability, the Wine Fest was, overall, better ranked than the Flower Fest.

## 4. Discussion

This exploratory study summarizes the physical and physiological effects of a custom-made VR exergame (FitFest) among older adults, as well as the system’s usability. The overall analysis indicates a prevalence of sedentary behavior, followed by light PA. The comparison between exergames revealed that the Wine Fest provided more PA intensity and a higher number of steps than the Flower Fest, although the differences were not statistically significant. Regarding the systems’ usability, both exergames were considered easy to use, and participants showed interest in playing them more often.

Following the literature, older adults are recommended to engage in at least moderate-intensity PA to achieve substantial health benefits [23], which was not accomplished by FitFest exergames. However, it is of note that light PA has been previously associated with physical health and well-being in the older adult population [30,31]. A detailed analysis of the gameplay must consider the inclusion of the tutorials and the rating of perceived exertion as important variables that have contributed to lower active time. Before each game scenario, a 30–40 s tutorial was available, during which the player had to replicate certain movements necessary to play. However, the time spent at this phase varied based on the player’s understanding, and sometimes extra verbal instruction was needed from the research team. The unfamiliarity with equipment, complex controls, and unclear instructions has been identified as a barrier for older adults in exergaming [32], underscoring the need for tutorial inclusion. Additionally, after each game scenario conclusion, users were asked to rate their perceived exertion using an interactive Borg scale, where they had to move laterally to choose the number that better reflected their effort. In some cases, players showed some difficulty in understanding this concept, demanding verbal instruction from the research team members. Finally, it is also important to mention that the last game scenario primarily stimulated cognitive abilities, with minimal physical movement (players only had to move to the sides to choose the puzzle piece they wanted), and therefore, it also contributed to sedentary behavior. Even though a significant part of gameplay has been spent in sedentary behavior, the literature has advocated that even small amounts of PA can benefit health [33,34]. On the other hand, it is of note that this is an exploratory study of a brand-new solution that can still be worked on.

The mean HR found in this study was slightly above the ranges reported in previous research on customized exergames targeting balance ability using Kinect-based motion-tracking (80–87 bpm) [35]. In contrast, a slightly higher mean HR was observed using Nintendo Wii Sports exergames (94 ± 10 bpm) [19]. However, previous studies used exergames targeting balance [35] and cardiorespiratory fitness [19] activities. Since FitFest exergames were developed to provide a multicomponent exercise program that incorporates strength, balance, agility, and aerobic capacity, the interpretation of mean HR should consider the combination of all activities performed during the game. Based on literature guidelines for PA in older adults, intensity should vary according to the targeted physical fitness components [34]. For instance, endurance exercise for older adults should correspond to a perceived exertion between 5 and 6 for moderate intensity and 7 to 8 for vigorous intensity, using a scale from 0 to 10 [34]. On the other hand, no specific guidelines have been designed for balance exercises regarding intensity [34]; however, considering the type of activities recommended, intensity should be lower than that of endurance exercises. Considering the different objectives set for each game scenario regarding the targeted physical fitness component, it would be expected to observe a fluctuation in intensity while exergaming, which influenced mean HR values.

In the meantime, the literature has reported that maximal HR indicates exercise intensity [36]. Using the age-predicted maximal HR equation proposed (220-age) [37,38], the mean maximal HR of the sample in this study was 153 bpm. Thus, the results of the mean HR observed during FitFest exergames (92.1 bpm) are equivalent to approximately 60% of the maximal HR, indicating light PA. Although the age-predicted maximal HR equation has presented some limitations, as it underestimates it in older adults [39], it is still an acceptable and widely used method in clinical settings [38].

The analysis of RPE scores provides valuable information regarding users’ perceived exertion in each scenario. The results suggest that tasks related to strength and aerobic capacity were perceived as more demanding, consistent with current guidelines [34]. Interestingly, the scenario dedicated to the cool-down phase, which focused exclusively on cognitive tasks, was perceived as moderate intensity. Previous research has suggested a relationship between self-efficacy and RPE among older adults [40]. Self-efficacy refers to an individual’s capacity to execute necessary behaviors to achieve specific performance outcomes [41]. Due to age-associated cognitive decline, cognitive tasks tend to be challenging for older adults [17]. In the current study, the challenge imposed by the memory and constructive skills activities presented might have influenced their perceived exertion. For this reason, it is crucial to consider the decrease in cognitive function related to age when proposing cognitive stimuli to older adults to avoid low self-efficacy levels, frustration, and demotivation.

Finally, the usability of the system analysis indicates that users perceived the exergames as easy to play and would like to use this tool more often, which is consistent with previous studies. In research examining the key barriers and facilitators of exergames played in the Xbox 360 (Microsoft, Redmond, WA, USA) with Kinect system, participants recognized and appreciated the potential of these games for increasing PA among a wide range of other benefits. From the participants’ perspectives, this system was seen as fun, helpful, cognitively stimulating, and beneficial for improving general health [24]. In another study, the authors described acceptable usability using the Active@Home exergame prototype (Active Assisted Living Programme, Brussels, Belgium) [42]. On the other hand, the results of the present study also showed that users agreed on the need for technical support while using the system. Indeed, older adults’ technical knowledge and experiences with new technologies are often restricted [42]. This underlines the importance of developing age-appropriate designs and flawless technical functionalities to guarantee usability [43]. Moreover, ensuring the presence of someone (i.e., staff members) to support using exergames and avoid barriers, including confusion and intimidation [24], seems imperative to ensure a safer, motivated, and more fun experience for this population.

This study has some limitations that should be mentioned. First, it is acknowledged that the relatively small sample size and the unequal number of sessions among participants may limit the statistical power and generalizability of our findings. However, it should be noted that the difficulty inherent in recruiting older adults for longitudinal technological interventions. Second, to assess the potential impact of FitFest on PA levels, physical fitness, and cognitive abilities, a higher number of sessions must be promoted by adopting a long-term implementation and including a control group. Third, all participants played FitFest exergames at the same difficulty level (standard level) to ensure a standardized protocol, which might not correspond to their actual physical and cognitive capabilities. Consequently, HR values could be lower than those recorded using a more individualized approach. Therefore, an initial physical and cognitive assessment is crucial for the future implementation of FitFest, as it will provide participants with a stimulus (difficulty level) more closely approximating their individual profiles. Finally, since FitFest was specifically designed based on Portuguese festivities, its suitability for other populations must be investigated.

Even though the current study brings essential insights on a multicomponent VR custom-made exergame system’s ability to promote light PA, which has been previously related to physical health and well-being in older adults [30,31]. Although sedentary behavior was significant during gameplay, indicating the need for future prototype adjustments, FitFest may be a complementary tool to traditional exercise programs for light PA promotion among older adults. Additionally, this innovative system was considered easy to use, and participants reported that they would like to use it more often, which emphasizes the potential of future exergame implementation to foster health. Finally, exergame interventions among older adults should consider the need for technical support to ensure safer participation and avoid demotivation that might arise from the target population’s limited knowledge of technology usage.

## 5. Conclusions

The current study describes and assesses a VR custom-made prototype (FitFest) composed of two exergames (Wine Fest and Flower Fest) designed to deliver a full exercise session to older adults. Sedentary behavior, followed by light PA, prevailed during gameplay, indicating that although there is room for future prototype adjustments to fulfill the objectives defined, FitFest can emerge as a complementary tool to traditional exercise interventions for light PA promotion among older adults. The scenarios targeting strength and aerobic tasks were perceived as the more demanding. In the overall system usability analysis, participants reported that the system was easy to use, although they would need technical support, which reinforces the need to develop age-appropriate designs and flawless technical functionalities to ensure usability. As a preliminary study on the physical and physiological characterization of a custom-made prototype, future research, including a control group and a larger sample size, is recommended to thoroughly investigate the physical and physiological responses elicited during gameplay.

## Figures and Tables

**Table 1:** Description of actions/movements performed on each scenario of the Wine Fest exergame.

Wine Fest							
Scenarios	Physical Fitness					Cognitive Abilities	
	Balance	Agility	Cardiorespiratory	Mobility	Strength	Memory	Constructive skill
Grape picking	Steps Grape picking	Side movements	Steps height Number of steps	Grape picking	Grape storage (lower body) Number of steps (lower body)	Grape’s color	None
Grape selection	Grape picking	Side movements	Speed of movement	None	None	Grape’s color	None
Grape transportation	Equipment control	Side movements (avoid obstacles)	Speed of movement	None	Preparing the transport (lower body) Equipment control (upper body)	Grape’s color	None
Winemaking	Steps	Side movements	Steps height Number of steps	None	Number of steps (lower body)	None	None
Poster session	None	None	None	Puzzle piece selection	None	Puzzle piece selection	Puzzle piece selection

**Table 2:** Description of actions/movements performed on each scenario of the Flower Fest exergame.

Flower Fest							
Scenarios	Physical Fitness					Cognitive Abilities	
	Balance	Agility	Cardiorespiratory	Mobility	Strength	Memory	Constructive skill
Flower picking	Steps Flower picking	Side movements	Steps height Number of steps	Flower picking	Flower storage (lower body) Number of steps (lower body)	Flower’s color	None
Flower carpet decoration	Flower picking	Side movements	Speed of movement	None	Flower storage (lower body)	Flower’s color	None
Flower festival (dance)	Feet and arms coordination	None	Speed of movement	None	Arm movements (upper body) Leg movements (lower body)	Movement sequence	None
Flower parade	Side steps	Side movements (avoid obstacles)	Speed of movement	None	Number of steps (lower body)	None	None
Poster session	None	None	None	Puzzle piece selection	None	Puzzle piece selection	Puzzle piece selection

**Table 3:** Descriptive statistics for PA intensity and HR while playing the FitFest exergames.

Variable	Mean ± Standard Deviation
Sedentary behavior (%)	56.5 ± 20.4
Light PA (%)	42.1 ± 19.3
Moderate PA (%)	1.4 ± 2.4
Vigorous PA (%)	0.1 ± 0.1
Moderate-to-vigorous PA (%)	1.4 ± 2.4
Total steps (*n*)	436.7 ± 308.7
Steps per minute (*n*)	10.8 ± 7.9
Average HR (bpm)	92.1 ± 14.5
Minimum HR (bpm)	71.8 ± 12.0
Maximum HR (bpm)	115.0 ± 25.3

PA (physical activity), HR (heart rate).

## Data Availability

The data supporting this study’s findings are available from the corresponding author upon reasonable request. The data are not publicly available due to privacy and ethical restrictions.
